# Synchronous Primary Vulvar Squamous Cell Carcinoma and Ciliated Cell Variant of Endometrioid Adenocarcinoma Arising in an Endometrial Polyp: A Rare Dual Malignancy

**DOI:** 10.7759/cureus.89917

**Published:** 2025-08-12

**Authors:** Kavita Mardi, Kanav Goyal, Arnav Jagota, Uday P Singh, Sahibjot S Romana

**Affiliations:** 1 Pathology, Indira Gandhi Medical College, Shimla, IND

**Keywords:** ciliated cell variant, endometrial polyp, endometrioid carcinoma, postmenopausal, synchronous malignancy, vulvar squamous cell carcinoma

## Abstract

Vulvar carcinoma is an uncommon gynecological malignancy predominantly affecting older women, with vulvar squamous cell carcinoma (VSCC) representing the most common subtype. Endometrial polyps are common benign uterine lesions, although a small proportion may harbor malignant transformation, most often to endometrioid adenocarcinoma. The ciliated cell variant of endometrioid adenocarcinoma (CCVEA) is an exceptionally rare subtype, characterized by neoplastic glands lined by ciliated cells and an indolent clinical course with a favorable prognosis. We report an extremely rare case of CCVEA arising within an endometrial polyp in a 68-year-old postmenopausal woman, who was concurrently diagnosed with VSCC. The patient presented with chronic genital pruritus and a progressively enlarging vulvar lesion. Hysteroscopic evaluation incidentally revealed three endometrial polyps, all of which were removed via polypectomy. Microscopic examination of the polypoidal lesion demonstrated widespread complex endometrial hyperplasia with marked atypia, primarily featuring a lining of ciliated epithelial cells, confirming FIGO (International Federation of Gynecology and Obstetrics) grade IA CCVEA. Concurrent vulvar biopsy showed human papillomavirus (HPV)-independent, well‑differentiated squamous cell carcinoma with prominent keratinization and keratin pearls, staged as FIGO grade II VSCC. The patient underwent radical anterior vulvectomy with distal urethrectomy and meatoplasty, bilateral inguinofemoral lymphadenectomy, exploratory laparotomy, extrafascial hysterectomy with bilateral salpingo‑oophorectomy, and bilateral pelvic lymph node sampling. All lymph nodes were negative for metastasis. At seven months postoperatively, the patient remains disease‑free. This case underscores the risk of synchronous gynecological malignancies and the diagnostic challenges involved in confirming two concurrent primary tumors. It also emphasizes the importance of comprehensive evaluation in postmenopausal women, who may remain asymptomatic despite harboring malignant endometrial polyps. This report presents a rare occurrence of dual primary malignancies in the female genital tract and offers valuable insight that may help guide future treatment protocols.

## Introduction

Vulvar cancer accounts for 5-8% of all gynecological malignancies, and vulvar squamous cell carcinoma (VSCC) is by far the most common histologic subtype, primarily affecting postmenopausal women. Established risk factors include human papillomavirus (HPV) infection, tobacco use, chronic inflammatory conditions of the vulva (e.g., lichen sclerosus), and states of immunosuppression such as human immunodeficiency virus (HIV) infection. Vulvar malignancies often present with chronic itching, bleeding, and unusual genital discharge, and can sometimes appear as raised nodules or ulcerated lesions. Definitive diagnosis requires biopsy of all suspicious vulvar lesions, and lymph node status should be evaluated radiologically. Histologically, these range from HPV‑related, p16‑positive warty carcinoma to HPV‑independent, p16‑negative keratinizing carcinoma. Management consists of primary surgical excision with histologically clear margins and tailored inguinofemoral lymph node assessment (sentinel‑node biopsy or lymphadenectomy), supplemented by adjuvant radiotherapy or chemoradiotherapy for high‑risk or advanced‑stage disease, with structured surveillance and long‑term follow‑up for early detection of recurrence [1].

Endometrial polyps are common benign growths in postmenopausal women; however, a recent study reported a malignant transformation rate of 2-3% [2]. The incidence of malignant conversion is higher in women who are postmenopausal, particularly those with large polyps, increased endometrial thickness, and coexisting risk factors such as obesity, diabetes, and hypertension [2-6]. Most documented malignant transformations give rise to low‑grade endometrioid adenocarcinoma, whereas variants such as clear cell or serous adenocarcinoma, which typically follow a more aggressive course, are less common [3-6]. The ciliated cell variant of endometrioid adenocarcinoma (CCVEA) is exceptionally rare, with only sporadic reports in the medical literature [7-12]. Consequently, our case represents a particularly unusual instance of CCVEA originating within an endometrial polyp.

In 1983, Hendrickson and Kempson first described 10 cases of the CCVEA in a retrospective review of 400 endometrial carcinoma specimens, identifying 10 adenocarcinomas with predominant ciliated cells (more than 75%) [8]. All 10 patients were postmenopausal, four were over 70 years of age, and each tumor was confined to the uterus. Residual ciliated adenocarcinoma was present in all resected uteri after biopsy, and in five cases, the carcinoma invaded the myometrium. In one patient, a microscopic focus of conventional endometrioid carcinoma, considered a simultaneous primary neoplasm, was also found in the ovary. Although ciliated cells are typically associated with benign endometrial conditions such as tubal metaplasia, this study highlights that, in rare instances, carcinomas - some with myometrial invasion - can also be composed predominantly of ciliated cells. While ciliated cells in endometrial biopsies are most often attributed to tubal metaplasia [8], that commonality should not preclude consideration of a ciliated carcinoma.

Patients with endometrial cancer most commonly present with abnormal uterine bleeding [2,3]. However, as in our case, they may be asymptomatic, with malignant polyps discovered incidentally [2,3]. Radiological imaging, hysteroscopic evaluation, and histopathological examination remain the cornerstones of diagnosis [4-7,13,14]. Histologically, CCVEAs are characterized by neoplastic glands lined predominantly by ciliated cells [7-13]. Standard management comprises total abdominal hysterectomy with bilateral salpingo‑oophorectomy and bilateral pelvic lymph node assessment to evaluate for metastatic spread [7,14].

Synchronous primary gynecological malignancies, such as concurrent ovarian and endometrial carcinomas, are frequently encountered, whereas synchronous tumors of the endometrium and cervix are rare [15]. However, the simultaneous occurrence of vulvar and endometrial carcinoma is exceptionally uncommon, and no large-scale series have been published to date. Likewise, CCVEA is exceedingly rare, with only isolated case reports and small series reported in the literature [7-12]. We herein present the case of a 68‑year‑old postmenopausal woman diagnosed concurrently with VSCC and CCVEA arising from an endometrial polyp. This case underscores the diagnostic challenges of identifying dual primary malignancies and the importance of confirming their independent origin. It also highlights the need for thorough evaluation of both vulvar lesions and endometrial polyps in postmenopausal women, who may remain asymptomatic despite harboring malignancy. Additionally, it emphasizes that CCVEA can be mistaken for tubal metaplasia, particularly on endometrial biopsy.

## Case presentation

A 68‑year‑old obese and postmenopausal woman presented with a one‑year history of persistent genital pruritus and, over the past three months, noted a growing lesion on the right vulva at the site of itching. Initially measuring 1×1 cm, the lesion had enlarged to 3×2 cm. She denied vaginal bleeding, discharge, abdominal or genital pain, and dysuria. There was no significant family history. On examination, there was an ulceroproliferative mass measuring 3×2 cm on the right labia majora, involving the labia minora and clitoris, and extending medially to the external urethral meatus. The cervix and vaginal walls were flushed, although a circumferential fibrotic band was noted at the 6 o’clock position in the mid‑vagina. No inguinal lymphadenopathy was detected.

**Figure 1:**
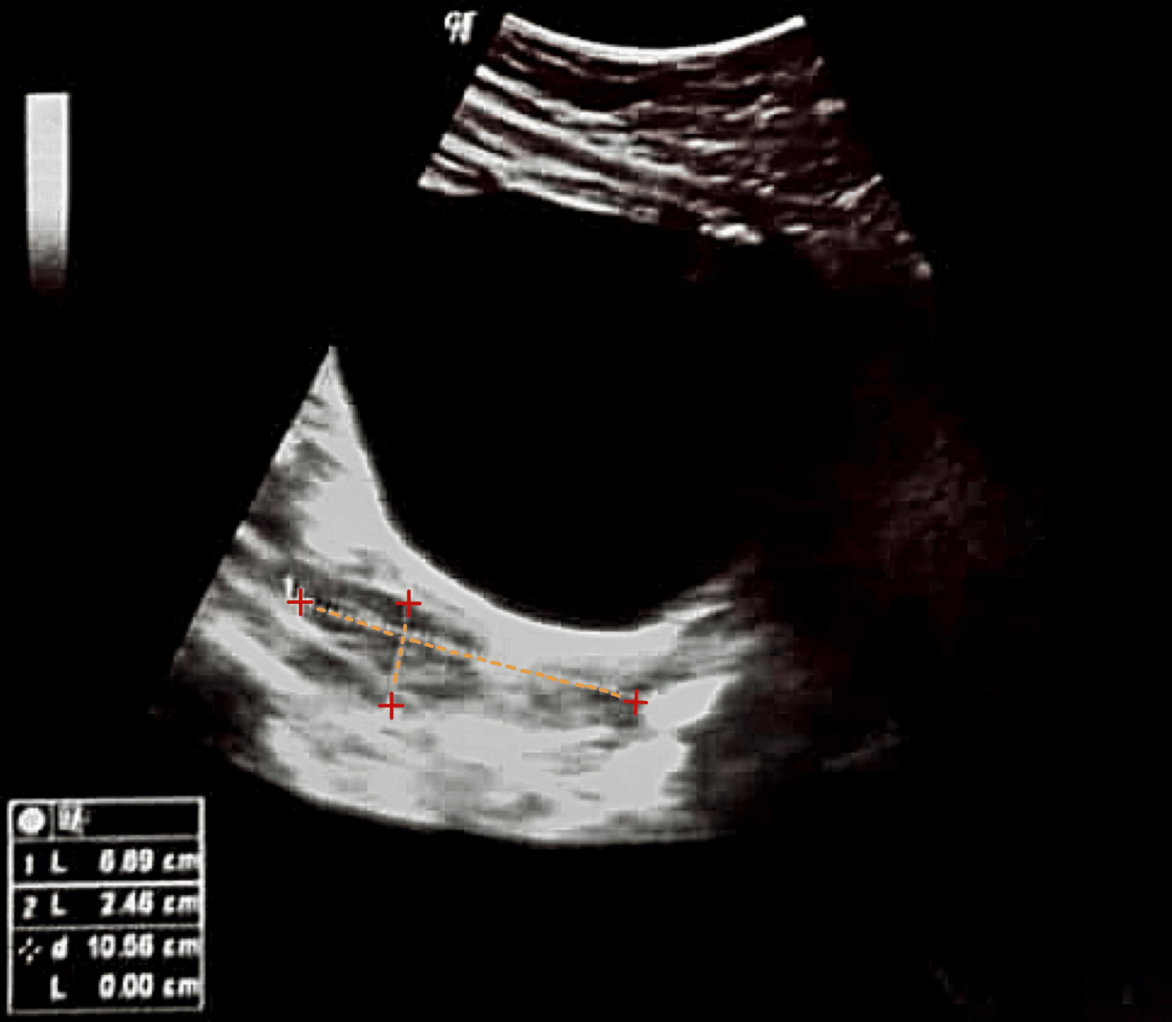
Transvaginal ultrasound of the endometrial lesion showing increased endometrial thickness.

Pelvic ultrasonography demonstrated a heterogeneously hypoechoic intramural lesion measuring 8×9 mm in the right anterior uterine wall, consistent with a subserosal fibroid. Bilateral adnexa appeared normal. The endometrial thickness measured 16.6 mm, significantly above normal for a postmenopausal woman, prompting the need for hysteroscopy (Figure 1).

Hysteroscopic evaluation showed two polyps measuring 3×2 cm arising from the left posterolateral surface of the endometrial cavity, along with another polyp measuring 2×1 cm located in the fundal region. The remaining endometrial cavity appeared atrophic. Endometrial curettage and polypectomy were performed, and multiple grey-white to grey-brown soft tissue fragments were sent for histopathological examination. A punch biopsy of the vulvar lesion was performed and submitted for histopathological evaluation.

**Figure 2:**
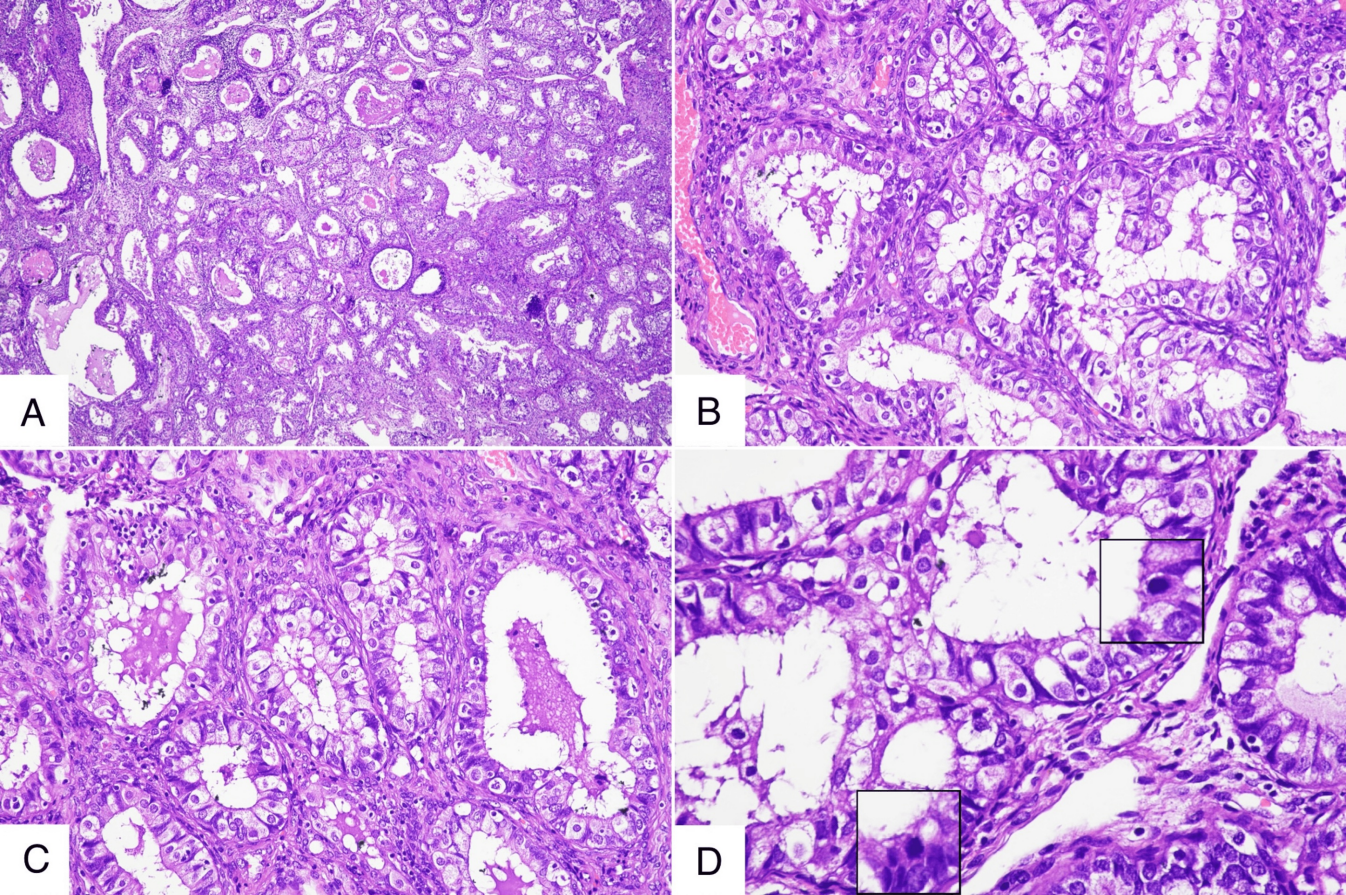
Histopathology of ciliated cell variant endometrioid adenocarcinoma arising in an endometrial polyp. (A) Low‑power view showing a fragmented endometrial polyp composed of crowded, back‑to‑back glands with focal stromal invasion. (B) Intermediate‑power view showing neoplastic columnar glands lined by abundant, fine apical cilia, a hallmark of the ciliated‑cell variant. (C) High-power view showing numerous hair‑like ciliary projections along the luminal border of the atypical glandular epithelial cells. (D) Higher magnification detail showing atypical mitotic figures within the glandular epithelium.

Histopathology showed multiple polypoidal endometrial fragments exhibiting features of a hyperplastic polyp. In several focal areas, closely packed, back-to-back arranged glands were seen with minimal intervening stroma and focal cribriform architecture. The glands were lined by cuboidal to columnar cells showing nuclear pleomorphism, enlarged round to ovoid vesicular nuclei, variably prominent nucleoli, and abundant light eosinophilic cytoplasm. The majority of the tumor cells exhibited cilia along their luminal borders, and eosinophilic secretions were observed within the glandular lumina (Figures 2A-2C). Frequent mitotic figures were also noted (Figure 2D). The intervening stroma displayed a desmoplastic response. These findings were consistent with a diagnosis of endometrioid carcinoma (ciliated cell variant), FIGO (International Federation of Gynecology and Obstetrics) grade IA, arising in an endometrial polyp.

**Figure 3:**
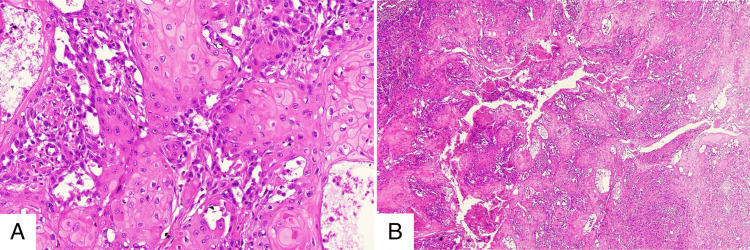
Histopathology of the primary well-differentiated vulvar squamous cell carcinoma. (A) High-power view showing atypical squamous cells with eosinophilic cytoplasm and prominent keratin pearl formation. (B) Low-power view showing an infiltrative growth pattern of malignant squamous cells invading the dermis, accompanied by a desmoplastic stromal reaction.

The vulvar biopsy specimen on immunohistochemistry revealed negative p16. Histopathological analysis of the vulvar biopsy revealed tumor cells displaying mild-to-moderate nuclear atypia, with enlarged, hyperchromatic nuclei, low mitotic activity, and minimal pleomorphism, as well as prominent keratinization, including dyskeratotic cells and well‑formed keratin pearls, confirming an HPV-independent, well‑differentiated VSCC (Figures 3A, 3B).

The planned surgical management included a radical partial anterior vulvectomy, distal urethrectomy with meatoplasty, bilateral inguinofemoral lymphadenectomy (saphenous vein-sparing), exploratory laparotomy, extrafascial hysterectomy with bilateral salpingo-oophorectomy, and bilateral pelvic lymph node sampling. Histopathological evaluation of all sampled lymph nodes revealed no evidence of metastasis. The stage of endometrial carcinoma was determined to be T1aN0M0, and the stage of vulvar cancer was T2N0M0. At the three- and six-month reviews, the patient had healed well, showed no evidence of recurrence on examination or imaging, and had returned to normal activities.

## Discussion

Vulvar cancer follows two distinct epidemiologic and etiologic pathways: an HPV-associated form, which is typically seen in younger women, and an HPV-independent variant, which often arises in the context of risk factors such as tobacco use, autoimmune disorders, HIV coinfection, and chronic vulvar dermatoses such as lichen sclerosus. The HPV-independent subtype is generally observed in older women and is associated with more aggressive clinical behavior compared to its HPV-associated counterpart. VSCC is the most common histological subtype of vulvar cancer, with approximately two-thirds of cases arising via the HPV-independent pathway. Histopathologically, HPV-independent VSCC is typically of the keratinizing type, characterized by keratin pearl formation and a high proportion of differentiated tumor cells. In contrast, basaloid and warty subtypes are more frequently associated with HPV-related disease. VSCC typically presents with long‑standing pruritus, bleeding, abnormal genital discharge, and vulvar ulceration. Accurate staging through vulvar biopsy and determination of HPV association are essential for appropriate diagnosis and treatment planning. Surgical resection remains the primary treatment modality, including wide local excision, radical vulvectomy, and lymph node assessment (via sentinel lymph node biopsy or bilateral inguinofemoral lymphadenectomy), depending on tumor size, depth of invasion, and risk of nodal metastasis [1]. In our patient, a one-year history of persistent pruritus led to the identification of a 3×2 cm ulceroproliferative lesion on the right labia majora. Biopsy confirmed an HPV-independent, well-differentiated VSCC, with HPV status verified using p16 immunohistochemistry. Although clinical examination revealed no inguinal lymphadenopathy, bilateral inguinofemoral lymphadenectomy was performed due to the documented risk of nodal metastasis in such tumors [1]. Additionally, urethrectomy was undertaken because of involvement of the distal urethra. The tumor was staged as T2N0M0.

Endometrial polyps are benign lesions, commonly seen in postmenopausal women and carry a 2.75% risk of malignant transformation, most often to endometrioid adenocarcinoma [2,6]. Additionally, 1.8% may exhibit atypical hyperplasia and 5.2% show hyperplasia without atypia, according to a recent systematic review and meta-analysis of 11,204 patients with endometrial polyps by Al-Rayes et al. [2]. The risk of malignant transformation is higher in postmenopausal women, particularly those with abnormal uterine bleeding, large polyps, increased endometrial thickness, or coexisting risk factors such as obesity, diabetes, and hypertension [2-6]. These patients may be symptomatic with abnormal uterine bleeding or may remain asymptomatic [2,3]. In our patient, the primary symptom was vulvar itching due to VSCC. She did not report any symptoms attributable to the endometrial polyps or carcinoma, underscoring the silent nature of such lesions, even when malignant. This incidental finding significantly altered the management plan, necessitating additional surgical intervention to address both malignancies. In a study by Ferrazzi et al. involving 1,152 asymptomatic women harboring endometrial polyps, 1,134 were found to have benign polyps, 14 had precancerous lesions, and only 4 (0.3%) had malignant lesions, suggesting the possibility of malignant endometrial polyps being asymptomatic [3]. Hysteroscopic polypectomy followed by histopathological examination is the recommended diagnostic and therapeutic approach, especially in high-risk patients [2-5,14]. In this case, during staging evaluation of vulvar cancer, pelvic ultrasonography revealed an endometrial thickness of 16.6 mm, and hysteroscopy identified three polyps, which were subsequently excised and submitted for histopathological examination. Incidentally, histological examination showed back-to-back ciliated glands with mild atypia, frequent mitoses, and desmoplastic stroma, confirming FIGO grade IA endometrioid carcinoma, ciliated cell variant [7-12]. It typically carries a favorable clinical outcome. Histological evaluation of resected polyps is crucial, even when clinical suspicion is low, as demonstrated by our case of adenocarcinoma identified in a polyp. Surgical management included extrafascial hysterectomy with bilateral salpingo-oophorectomy and bilateral pelvic lymph node sampling to assess tumor spread. The final stage of the tumor was T1aN0M0.

The CCVEA is exceedingly rare and, to the best of our knowledge, has not been previously reported arising from an endometrial polyp [7-12]. CCVEA is characterized by neoplastic glands predominantly lined by ciliated epithelial cells [7-13]. Cilia are specialized extensions of epithelial cells found in various tissues, functioning primarily in movement and sensory perception. They are classified into two main types: motile cilia and non‑motile (primary) cilia. Motile cilia are typically located in the respiratory tract, lungs, and middle ear, where they help move fluids or particles across epithelial surfaces. In contrast, primary cilia are involved in sensory functions and are especially important in organs such as the kidney and in retinal photoreceptors. Structurally, cilia consist of a core of microtubules enclosed by the plasma membrane. Motile cilia exhibit a (9+2) arrangement, with nine outer pairs of microtubules surrounding two central ones, whereas primary cilia have a (9+0) configuration, lacking the central pair. Both types are anchored to the cell by a basal body located in the cytoplasm [7]. Ciliated cells are the hallmark of CCVEA but may cause confusion with benign tubal metaplasia on limited endometrial biopsies [7-9,11-13]. In the endometrium, ciliated cells most commonly signify benign processes, particularly tubal metaplasia [7]. However, CCVEA can be distinguished by the presence of ciliated glands showing cytological atypia, and by evidence of myometrial or vascular invasion [7,9,11]. Moreover, because the ultrastructure of cilia in malignant glands is identical to that of non‑neoplastic epithelium [7,11], reliance on limited sampling alone risks under‑recognition of this rare carcinoma [7,9,11].

The synchronous occurrence of vulvar carcinoma and endometrioid adenocarcinoma arising in an endometrial polyp is exceptionally rare, with no prior case series; moreover, CCVEA arising in an endometrial polyp has never been reported. Synchronous tumors must be recognized as independent primary neoplasms, rather than metastatic lesions of each other, to avoid misclassification. When two malignancies occur at the same time in the genital tract, it is critical to determine whether each represents an independent primary cancer or whether one has metastasized to a second site. If a vulvar tumor, for example, was actually a metastasis from an endometrial carcinoma, it would automatically be classified as FIGO stage IV endometrial cancer, indicating distant spread and carrying a much poorer prognosis. Conversely, if the endometrial lesion was assumed to be metastatic from the vulva, it would be staged as FIGO stage IV vulvar cancer. Histopathological evaluation is therefore essential to confirm distinct tumor origins, establish synchronicity, ensure accurate diagnosis, and avoid unnecessary radical or overtreatment. To diagnose synchronous tumors, the following criteria should be fulfilled: (a) each lesion exhibits unequivocal malignant histology, (b) the tumors are histologically different from one another, and (c) metastatic spread between them is excluded [15]. In our patient, all three criteria were met. Although synchronous gynecological malignancies are uncommon, their coexistence poses unique diagnostic and therapeutic challenges. Management requires a multidisciplinary approach, tailoring treatment to each tumor’s characteristics, patient age, histologic subtype, stage, grade, and lymphovascular invasion [15]. Because no standardized protocols exist for concurrent endometrial adenocarcinoma and vulvar carcinoma, we integrated the standard treatment strategies for both entities simultaneously in this case. Typically, synchronous tumors are definitively diagnosed only after surgical resection, with each malignancy managed according to its respective guidelines. However, in our case, the simultaneous nature of both tumors was established preoperatively, allowing for a single, coordinated treatment plan. Managing synchronous VSCC and endometrial carcinoma required radical partial anterior vulvectomy, distal urethrectomy with meatoplasty, bilateral inguinofemoral lymphadenectomy (saphenous vein-sparing), exploratory laparotomy, extrafascial hysterectomy with bilateral salpingo-oophorectomy, and bilateral pelvic lymph node sampling [1,14]. All sampled lymph nodes showed no evidence of tumor spread. The final staging was T2N0M0 for VSCC and T1aN0M0 for endometrioid carcinoma confined to a polyp; both carry a favorable prognosis, with five-year survival rates exceeding 80% [1,14]. The outlook for patients with synchronous vulvar carcinoma and endometrial adenocarcinoma remains undetermined; however, synchronous tumors generally have a worse prognosis than solitary primaries, yet they have better survival than metastatic disease [15]. In the absence of robust data, follow‑up should include regular pelvic and rectal examinations and vigilant surveillance for recurrence or metastasis.

## Conclusions

In conclusion, this case highlights the uncommon coexistence of vulvar and endometrial carcinomas in a postmenopausal woman and the extraordinarily rare occurrence of the CCVEA arising within an endometrial polyp. Moreover, it reinforces that endometrial polyps, even when asymptomatic, can harbor malignancy, underscoring the importance of histopathological examination of all excised gynecological specimens. Pathologists should remain alert to the possibility of CCVEA, as its ciliated architecture can mimic benign tubal metaplasia, especially on limited biopsies in postmenopausal patients. While tubal metaplasia is the most common ciliated lesion of the endometrium, it should not preclude careful evaluation for ciliated carcinoma. Equally significant is distinguishing synchronous primary tumors from metastatic disease, which requires comprehensive imaging and meticulous histological review to confirm their independent origins. This case underscores the need for vigilance when evaluating vulvar lesions and endometrial polyps, as synchronous malignancies may be overlooked without thorough assessment. Given the complexity of managing synchronous gynecological cancers and the absence of standardized treatment protocols for concurrent VSCC and CCVEA, a multidisciplinary surgical approach tailored to both tumor sites is essential. Finally, vigilant long-term follow-up is critical to detect any local recurrence or distant metastasis. By documenting this unique dual malignancy, we contribute to the understanding of its biological behavior and help lay the foundation for future treatment guidelines.
